# A New Species of *Scymnus* (Coleoptera, Coccinellidae) from Pakistan with Mitochondrial Genome and Its Phylogenetic Implications †

**DOI:** 10.3390/insects15050371

**Published:** 2024-05-19

**Authors:** Zafar Iqbal, Rashid Azad, Xiao-Sheng Chen, Xiao-Ling Lin, Zichen Zhou, Xing-Min Wang, Rui-E Nie

**Affiliations:** 1 Key Laboratory of the Conservation and Exploitation of Biological Resources, College of Life Sciences, Anhui Normal University, Wuhu 241000, China; iqzafarshah@gmail.com (Z.I.); linxiaooling@163.com (X.-L.L.); 2 Department of Entomology, The University of Haripur, Haripur 22620, Pakistan; rashidazad@uoh.edu.pk; 3 Department of Entomology, South China Agricultural University, Guangzhou 510640, China; 4 Engineering Research Center of Biological Control, Ministry of Education, Guangzhou 510642, China; xshchen@scau.edu.cn; 5 Department of Forest Protection, College of Forestry and Landscape Architecture, South China Agricultural University, Guangzhou 510642, China; 6 Department of Life Sciences, Imperial College London, Exhibition Road, London SW7 2BX, UK; z.zhou@nhm.ac.uk; 7 Department of Life Sciences, Natural History Museum, Cromwell Road, London SW7 5BD, UK; 8 Engineering Technology Research Center of Agricultural Pest Biocontrol of Guangdong Province, Guangzhou 510640, China

**Keywords:** Coccinellidae, Scymnini, *Scymnus*, *Pullus*, new species, mitochondrial genome, Pakistan

## Abstract

**Simple Summary:**

The study presents the new species *Scymnus* (*Pullus*) *cardi* sp. nov. and provides its mitochondrial genome. We describe the distinguishing features of *S*. (*P*.) *cardi* when compared with related species and discuss its habitat and feeding preferences. Additionally, based on the mitochondrial genomic dataset, the phylogeny of Coccinellidae is analyzed. The results confirm the position of the new species, which is nested within the genus *Scymnus*, and recovered subfamilies as monophyletic groups (such as Coccinellinae and Microweiseinae). These findings contribute to the understanding of Coccinellidae evolution and highlight the need for further taxonomic and genetic studies within the family.

**Abstract:**

In this study, a new species of the subgenus *Pullus* belonging to the *Scymnus* genus from Pakistan, *Scymnus (Pullus) cardi* sp. nov., was described and illustrated, with information on its distribution, host plants, and prey. Additionally, the completed mitochondrial genome (mitogenome) of the new species using high-throughput sequencing technology was obtained. The genome contains the typical 37 genes (13 protein-coding genes, two ribosomal RNAs, and 22 transfer RNAs) and a non-coding control region, and is arranged in the same order as that of the putative ancestor of beetles. The AT content of the mitogenome is approximately 85.1%, with AT skew and GC skew of 0.05 and −0.43, respectively. The calculated values of relative synonymous codon usage (RSCU) determine that the codon UUA (L) has the highest frequency. Furthermore, we explored the phylogenetic relationship among 59 representatives of the Coccinellidae using Bayesian inference and maximum likelihood methods, the results of which strongly support the monophyly of Coccinellinae. The phylogenetic results positioned *Scymnus (Pullus) cardi* in a well-supported clade with *Scymnus (Pullus) loewii* and *Scymnus* (*Pullus*) *rubricaudus* within the genus *Scymnus* and the tribe Scymnini. The mitochondrial sequence of *S*. (*P*.) *cardi* will contribute to the mitochondrial genome database and provide helpful information for the identification and phylogeny of Coccinellidae.

## 1. Introduction

*Scymnus* is one of the largest genera of ladybird beetles (Coleoptera, Coccinellidae, Scymnini), comprising more than 800 described species. This genus is composed of eight subgenera: *Scymnus* Kugelann, *Pullus* Mulsant, Didion Casey, *Neopullus* Sasaji, *Parapullus* Yang, *Mimopullus* Fürsch, *Orthoscymnus* Canepari, and *Canalipullus* Lafer [1,2]. Many taxonomists, such as Crotch [3], Sicard [4], Korschefsky [5], Kamiya [6], Sasaji [7], Gordon [8], Kovář [9], Ślipiński [10], Giorgi et al. [11] and Seago et al. [12], have established different classification systems for this genus. Recently, Che et al. [13] have classified it into the tribe Scymnini of the subfamily Coccinellinae.

*Pullus* has been established by Mulsant [14] as a subgenus of *Scynmus*, with *Coccinella subvillosa* Goeze [15] as a type species, and this has been accepted by most authors [1,2,6,8,16,17,18,19]. Taxonomic identification at the species level of subgenus *Pullus* is difficult with external appearance due to its small size and polymorphism, therefore identification has relied on male genital characteristics. Based on the characteristics of male genitalia, Chen et al. [2] divided the subgenus *Pullus* into five groups: *hingstoni*, *subvillosus*, *impexus*, *perdere,* and *sodalis*. Members of this genus are known to be general predators of aphids, adelgids, mealybugs, and scale insects [20]. The nominative subgenus is characterized by the following combination of characteristics: antennae with 11 antennomeres (a short pedicle that is narrower than the scape); a prosternal process that is relatively narrow with convergent carinae, reaching to anterior margins; an abdomen with six ventrites; and the abdominal postcoxal line completed (a characteristic which separates it from the subgenus *Scymnus*).

The members of subgenus *Pullus* are widely distributed all over the world, with 461 species described from the palaearctic (149) [16], nearctic (154) [8,21], neotropical (27) [22], Australian (5) [23], oriental (78) [24,25,26] and afrotropical regions (50) [27,28,29,30]. Currently, six species of *Pullus* have been recorded from Pakistan.

In this paper, a new species, *Scymnus* (*Pullus*) *cardi* sp. nov., and a single new country record named *Scymnus* (*Pullus*) *latifolius* Poorani, 2018 of the group *perdere* are recorded from Pakistan. Here, we (i) describe this new species and compare its diagnostic characteristic with related species (*Scymnus* (*Pullus*) *latifolius*, (ii) analyze the mitochondrial genomic characteristics, and (iii) reconstruct the phylogenetic relationship of the family Coccinellidae.

## 2. Materials and Methods

### 2.1. Specimens and Taxonomy

The specimens were collected by sweeping net method in Pakistan and preserved in absolute ethanol at −20 °C. External morphology was observed with a Labomed microscope (CZM6). Male genitalia were dissected and cleared in 10% KOH solution by boiling several times and were then washed with 70% and 90% ethanol. Images were taken using a Nikon Digital camera (SMZ 1500) attached to the stereo microscope. Photographs were edited using Helicon Focus 8.1 (Helicon Soft Ltd., Kyiv, Ukraine.) and Adobe Photoshop 2020 (Adobe Inc., San Jose, CA, USA).

Measurements were made by using an ocular micrometer, which works at the millimeter (mm) scale. The following abbreviations were used: TL—total length from apical margin of clypeus to elytral apex; TW—total width across both elytra at widest part; TH—total height at highest part of elytra; HW—head width at widest part including eyes; PL—pronotal length from the middle of anterior margin to the base of pronotum; PW—pronotal width at widest part; EL—elytral length along suture from base to apex including scutellum.

Morphological terminology was determined following Ślipiński [10] and Chen et al. [1,2]. Type specimens were deposited at the Department of Entomology, South China Agricultural University, Guangzhou, China. Some paratypes were deposited at the Pir Mehr Ali Shah -Arid Agriculture University, Rawalpindi, Pakistan.

The following acronyms are used in this paper for the specimens’ depositories.
PMAS-AAU-Pir Mehr Ali Shah-Arid Agriculture University, Rawalpindi, Pakistan.SCAU-Department of Entomology, South China Agricultural University, Guangzhou, China.

### 2.2. DNA Extraction, Sequencing, Mitogenome Assembly, and Annotation

The genomic DNA was extracted from the selected adult specimens using the DNeasy Blood and Tissue Kit (Qiagen, Hilden, Germany) following the manufacturer’s protocol, eluted in 150 μL AE buffer, and kept at −20 °C until used. The mitochondrial genomes were sequenced using shotgun sequencing of total genomic DNA on the Illumina HiSeq. 2500 platform (University of Liverpool) with libraries having a 350 bp insert size and 250 bp paired-end sequencing. The Illumina sequencing library was prepared for a pool of 60 ladybird beetle samples. Initially, low-quality and short reads were removed using Prinseq [31], and the remaining high-quality reads were preliminarily assembled using SPAdes genome assembler v3.15.5, MEGAHIT v1.2.9, and MaSuRCA v4.0.5, respectively. K-mer sizes for SPAdes and MEGAHIT were set as 21, 33, 55, 77, 99, 127 and kept at the default level for MaSuRCA [32,33,34,35]. Based on the assembly results of the three assemblers above, the final contigs were assembled using Metassembler v1.5 [36]. Contigs from the mixed library were associated with a specific species by matching them to a shotgun-sequenced cox1 gene fragment that was used as bait and was obtained with primers Ill_B_F [37] and Fol_degen_rev [38]. Identification required a minimum of 99% identity in the Blast alignment.

Geneious Prime 2020.2.4 was used to perform gene annotations, check circularization, and extract the protein-coding gene and rRNAs from the mitochondrial genome [39]. A map of the mitogenomes was created using CGView Server (http://cgview.ca, visited on 25 January 2024) [40]. The base compositions of the mitochondrial genome were analyzed by MEGA v.11 [41]. Transfer RNAs (trnS1) composition and codon usage of the mitogenome of *Scymnus* (*Pullus*) *cardi* sp. nov. was compared with *Scymnus* (*Pullus*) *loewii*, (GenBank: MZ303019) and *Scymnus* (*Pullus*) *rubricaudus* (GenBank: MZ303020). The secondary structures and the anticodon of tRNAs of mitochondrial genomes were identified using MITOS Web Server (http://mitos2.bioinf.uni-leipzig.de/index.py (accessed on 15 May 2024)) [42] and tRNAscan-SE v1.3.1 [43]. The equations for computing AT-skew and GC-skew were as follows: GC-skew = [G% − C%]/[G% + C%] and AT-skew = [A% − T%]/[A% + T%] [44]. The codon and relative synonymous codon usage (RSCU) of 13 protein-coding genes (13 PCGs) were determined in Phylosuite [45,46]. The mitogenome of *Scymnus* (*Pullus*) *cardi* has been submitted to GenBank under Accession number PP639204, as shown in Table 1.

### 2.3. Phylogenetic Analyses

The phylogenetic position of *Scymnus* (*Pullus*) *cardi* and the high-level relationships of the family Coccinellidae were inferred from 59 Coccinellidae mitochondrial genome sequences, with *Dastarcus helophoroides*, *Discolomatinae* sp., and *Tenebrio molitor* used as outgroups (Table S1). The 13 protein-coding genes (PCGs) and 2 ribosomal RNAs (rRNAs) were extracted from each mitochondrial genome by Geneious Prime 2020.2.4 [39] and aligned separately using MUSCLE v.3.8 with default settings [47]. The phylogenetic analyses were carried out using the following two datasets: (1) 13 PCGs + 2 rRNAs, composed of the 13 protein-coding genes, including all codons and 2 ribosomal RNAs; (2) 13 PCGs_AA, wherein the 13 protein-coding genes were translated into amino acids. Gaps and ambiguous sites in the multiple alignment sequences were filtered using Gblocks 0.91 under default parameters [48]. The aligned gene datasets were then concatenated using SequenceMatrix v.1.9 [49]. The trees were inferred using PhyloBayes MPI v1.5a [50] and IQ-TREE v2.2.0 [51]. In the PhyloBayes analyses (all three datasets above) the CAT-GTR model was used. Two parallel and independent tree searches were performed until the discrepancies were lower than 0.1 (maxdiff less than 0.1). A consensus tree was computed using the remaining trees from two runs after the initial 25% of trees were discarded as burn-in. Maximum likelihood phylogeny was inferred using IQ–TREE v2.2.0. The MEP model was used for the bootstrapping phase, and node support in all ML analyses was calculated using 1000 SH-aLRT repetitions and 1000 UFBoot2 bootstraps (-B 1000, -alrt 1000), respectively [52].

**Table 1:** List of reference mitochondrial genomes chosen for phylogenetic analysis.

Family	Subfamily	Tribe	Species	Accession Numbers	Size bp	A + T%	AT% of All PCGs	References
Coccinellidae	Coccinellinae	Coccinellini	*Aiolocaria hexaspilota*	MK583344	17,549	80.2	79.7	[53]
	Coccinellinae	Coccinellini	*Anatis ocellata*	KX035143	17,092	79.3	78.2	Unpublished
	Coccinellinae	Coccinellini	*Anisosticta* *novemdecimpunctata*	KT876880	15,289	78.9	77.9	[54]
	Coccinellinae	Coccinellini	*Calvia championorum*	KX132085	17,575	78.2	77.3	Unpublished
	Coccinellinae	Coccinellini	*Calvia decemguttata*	KX087252	16,425	78.4	77.2	Unpublished
	Coccinellinae	Coccinellini	*Calvia muiri*	MF992928	17,126	78.3	77.2	[55]
	Coccinellinae	Coccinellini	*Cheilomenes sexmaculata*	MW845811	17,297	78	77.2	[56]
	Coccinellinae	Coccinellini	*Coccinella lama*	MW029464	18,932	77.1	77.2	[57]
	Coccinellinae	Coccinellini	*Coccinella septempunctata*	OU015583	19,413	76.3	77.1	Unpublished
	Coccinellinae	Coccinellini	*Coccinella transversoguttata*	OK624419	17,575	77.9	77.2	Unpublished
	Coccinellinae	Coccinellini	*Coleomegilla maculata*	KJ778881	17,516	77.5	75.8	[58]
	Coccinellinae	Coccinellini	*Cycloneda sanguinea*	KJ778883	15,118	79	77.6	[58]
	Coccinellinae	Coccinellini	*Cycloneda munda*	KJ778882	14,292	77.4	76.3	[58]
	Coccinellinae	Coccinellini	*Eriopis connexa*	MG253268	17,652	79.5	78.7	Unpublished
	Coccinellinae	Coccinellini	*Eriopis patagonia*	MN509443	15,720	80.1	79.2	[59]
	Coccinellinae	Coccinellini	*Harmonia axyridis*	KR108208	16,387	78.7	77.4	[60]
	Coccinellinae	Coccinellini	*Harmonia eucharis*	MW029462	17,441	76	74.9	[57]
	Coccinellinae	Coccinellini	*Harmonia quadripunctata*	KX087296	18,051	76.5	75.5	Unpublished
	Coccinellinae	Coccinellini	*Hippodamia convergens*	KX755331	18,419	78.4	77.2	Unpublished
	Coccinellinae	Coccinellini	*Hippodamia* *tredecimpunctata*	KJ778889	17,275	77.6	76.1	[58]
	Coccinellinae	Coccinellini	*Hippodamia undecimnotata*	KX087298	15,587	77.5	76.1	Unpublished
	Coccinellinae	Coccinellini	*Hippodamia variegata*	MK334129	17,823	77.5	76.6	[61]
	Coccinellinae	Coccinellini	*Lemnia saucia*	MK574678	14,106	78.9	78.1	[62]
	Coccinellinae	Coccinellini	*Megalocaria dilatate*	MZ983384	18,608	79.2	78.5	Unpublished
	Coccinellinae	Coccinellini	*Oenopia dracoguttata*	MW029467	19,220	78.1	77.3	[57]
	Coccinellinae	Coccinellini	*Oenopia sauzeti*	MW530420	17,630	80.1	78.2	Unpublished
	Coccinellinae	Coccinellini	*Oenopia formosana*	OR804096	17,885	79.1	77.9	Unpublished
	Coccinellinae	Coccinellini	*Olla v-nigrum*	MZ303015	14,448	76.9	75.6	Unpublished
	Coccinellinae	Coccinellini	*Propylea japonica*	KM244660	15,027	79.1	78.6	[63]
	Coccinellinae	Coccinellini	*Propylea* *quattuordecimpunctata*	MF992931	17,471	79.4	78.6	Unpublished
	Coccinellinae	Coccinellini	*Propylea* sp.	KX132084	15,915	79.6	78.7	Unpublished
	Coccinellinae	Coccidulini	*Cryptolaemus montrouzieri*	KT874575	17,010	79.5	78.4	Unpublished
	Coccinellinae	Coccidulini	*Coccidula rufa*	JX412767	10,589	76.9	76.6	Unpublished
	Coccinellinae	Chilocorini	*Chilocorus bipustulatus*	MN053054	12,229	79.1	78.9	[55]
	Coccinellinae	Chilocorini	*Chilocorus rubidus*	OQ130027	16,801	78.3	77.1	Unpublished
	Coccinellinae	Epilanchnini	*Afissula kambaitana*	MF992930	14,082	77.3	76.7	Unpublished
	Coccinellinae	Epilanchnini	*Epilachna admirabilis*	MN053053	17,445	79.9	78	[55]
	Coccinellinae	Epilanchnini	*Henosepilachna pusillanima*	KJ131489	16,216	78.2	76.8	[64]
	Coccinellinae	Epilanchnini	*Henosepilachna* *vigintioctopunctata*	MG584727	17,057	79.2	78.2	[55]
	Coccinellinae	Epivertini	*Epiverta chelonia*	ON209194	17,347	75.8	74.2	[65]
	Coccinellinae	Hyperaspini	*Brachiacantha groendali*	MZ303003	15,499	79.6	77.9	Unpublished
	Coccinellinae	Hyperaspini	*Hyperaspis festiva*	MZ303012	15,999	78.2	77.3	Unpublished
	Coccinellinae	Noviini	*Rodolia quadrimaculata*	MN053055	12,660	75.7	75.3	[55]
	Coccinellinae	Psylloborini	*Illeis bistigmosa*	MZ325765	17,840	77.5	76.6	[66]
	Coccinellinae	Psylloborini	*Illeis cincta*	MF992929	15,856	76.4	75.3	[55]
	Coccinellinae	Psylloborini	*Illeis koebelei*	OK012004	17,054	77.2	75.2	Unpublished
	Coccinellinae	Psylloborini	*Psyllobora lenta*	MZ303017	14,462	77.4	76.1	Unpublished
	Coccinellinae	Psylloborini	*Vibidia duodecimguttata*	MT114193	19,627	75.4	74.6	[67]
	Coccinellinae	Scymnini	*Nephus* (*Bipunctatus*) *includens*	MN164642	16,638	80.1	78.8	[68]
	Coccinellinae	Scymnini	*Nephus* (*Nephus*) *oblongosignatus*	MT445723	16,647	79.6	77.7	[68]
	Coccinellinae	Scymnini	*Nephus* (*Geminosipho*) *reunioni*	MN164643	17,031	79	77.1	[68]
	Coccinellinae	Scymnini	*Nephus* (*Nephus*) *apolonia*	MN164644	16,430	77.5	76.4	[68]
	Coccinellinae	Scymnini	*Nephus* (*Nephus*) *voeltzkowi*	MN164646	17,060	79.9	78.6	[68]
	Coccinellinae	Scymnini	*Scymnus* (*Pullus*) *cardi* sp. nov.	PP639204	15,416	78.5	77.2	THIS STUDY
	Coccinellinae	Scymnini	*Scymnus* (*Pullus*) *loewii*	MZ303019	15,629	79.4	78.4	Unpublished
	Coccinellinae	Scymnini	*Scymnus* (*Pullus*) *rubricaudus*	MZ303020	14,520	78.8	78.3	Unpublished
	Coccinellinae	Subcoccinellini	*Subcoccinella* *vigintiquattuorpunctata*	KT780695	14,645	76.1	75.2	Unpublished
	Microweiseinae	Microweiseini	*Coccidophilus cariba*	MN447521	15,343	78.3	77.4	[69]
Bothrideridae	Bothriderinae	Dastarcini	*Dastarcus helophoroides*	NC_024271	15,878	79.1	77.5	[70]
Discolomatidae	Discolomatinae	-	*Discolomatinae* sp.	JX412748	14,141	77.7	77	Unpublished
Tenebrionidae	Tenebrioninae	Tenebrionini	*Tenebrio molitor*	KF418153	15,785	72.4	69.3	[71]

## 3. Results and Discussion

### 3.1. Taxonomy

Genus *Scymnus* Kugelann, 1794

*Scymnus* Kugelann, 1794: 545 [72]. Type species: *Scymnus nigrinus* Kugelann, 1794 [72], subsequently designated by Westwood [73].

Distribution. Worldwide.

Subgenus *Pullus* Mulsant, 1846

Figure 1A–G

*Scymnus* (*Pullus*) Mulsant, 1846: 241 [14]. Type species: *Coccinella subvillosa* Goeze, 1777 [15], subsequently designated by Korschefsky [5].

**Figure 1:**
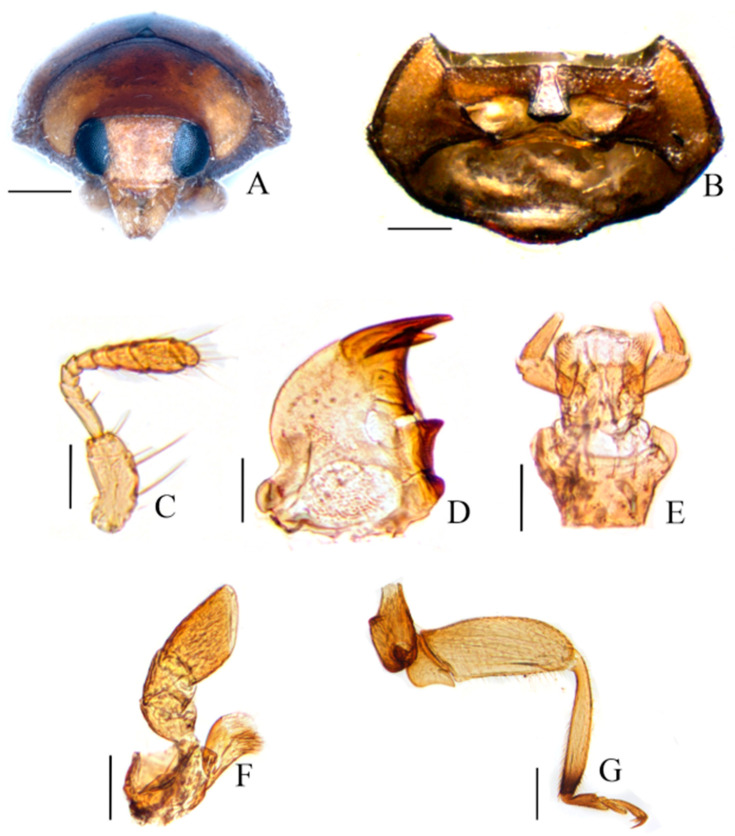
Characteristics of *Scymnus* (*Pullus*) *cardi* sp. nov. (**A**) Head. (**B**) Prosternum. (**C**) Antenna. (**D**) Mandible. (**E**) Labium. (**F**) Maxilla. (**G**) Hind leg. Scale bar (mm): (**A**–**F**) 0.15; (**G**) 0.5.

**Figure 2:**
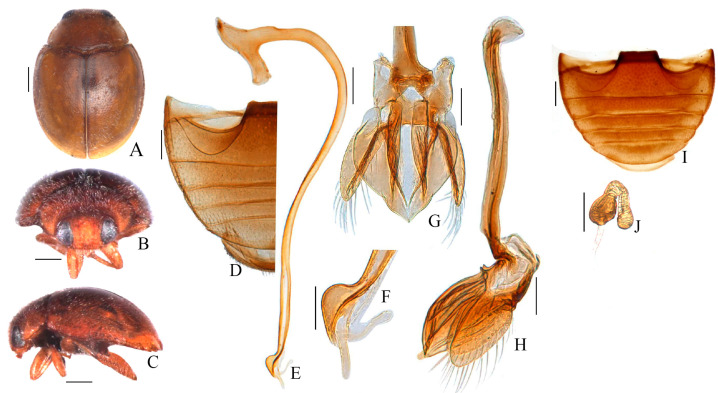
*Scymnus* (*Pullus*) *cardi* sp. nov. (**A**) Dorsal view. (**B**) Frontal view. (**C**) Lateral view. (**D**) Male abdomen. (**E**) Penis. (**F**) Apex of penis. (**G**) Ventral view of tegmen. (**H**) Lateral view of tegmen. (**I**) Female abdomen. (**J**) Spermatheca. Scale bar (mm): (**A**–**D**,**I**,**J**) 0.5; (**C**–**F**) 0.15.

Diagnosis. Subgenus *Pullus* Mulsant species can be easily distinguished from other subgenera of *Scymnus* by the following combination of characteristics: the body is oval or elongated oval and the head capsule is rectangularly oval and finely punctate (Figure 1A); the prosternal process has well-developed carinae, that are convergent and reach to the anterior margin (Figure 1B); the antenna are composed of 11 antennomeres (Figure 1C); the mandible has double teeth, with the outer tooth slightly longer than the inner tooth (Figure 1D); the labrum with apical palpomere is narrow and shorter than the penultimate one (Figure 1E); a maxilla with terminal palpomere securiform and obliquely truncate apical (Figure 1F); a tibia without spur and a tarsal claw that is bifid (Figure 1G); an abdomen with six ventrites and an abdominal postcoxal line that is complete at the first ventrite, or in the male the fifth and sixth ventrites, and is usually moderately or strongly emarginate apically (Figure 2B).

Distribution. Worldwide.

*Scymnus* (*Pullus*) *cardi* sp. nov.

Figure 2A–H

Type specimens. Holotype: male, Kot Sarang (33°1.540′ N, 72°24.212′ E, 477 m), Chakwal, Punjab, 06. viii.2016, Rashid A., leg (SCAU). Paratypes: Khyber Pakhtunkhwa: 1 male, Mardan, Mardan Rd. (34°4.077′ N, 72°15.233′ E, 322 M), 30.ix.2016, Rashid A., leg. (SCAU): Punjab: 2 males, 3 females, Chakwal, Kallar Kahar (Izhar Farm) (32°54.370′ N, 72°30.151′ E, 416 m), 20.viii.2017, Z. Iqbal leg. (PMAS-AAU).

Etymology. The name of this new species is derived from the Latin adjective cardio, meaning heart, referring to the penis guide as seen in the lateral view.

Description. TL: 1.40–1.60 mm; TW: 1.01–1.21 mm; TH: 0.64–0.77 mm; EL/EW: 1.02–1.03; TL/TW: 1.32–1.41; HW/PW: 0.62–0.67; PL/PW: 0.49–0.51.

The body (Figure 2A) is oval and convex, the dorsum surface is covered with dense pubescence. The head, mouthparts, and antennae are yellowish-brown. The pronotum, scutellum, elytra, and prothoracic hypomeron are brownish yellow. The elytral epipleuron is brown with dark brown outer and inner margins (Figure 2B,C). The under-side is dark brown. The leg (Figure 1G) is yellowish-brown, except for the apical part of the tibia, which is dark brown.

The head (Figure 2C) is small, 0.63 times the pronotal width, and has fine punctures that are 0.5–1.0 diameters apart. The eye facets are dense, with an interocular distance that is 0.45× the head width. The pronotum is 0.77× the elytral width (PW/EW = 0.85/1.11). The eyes are densely faceted, with sparse hairs, and an interocular distance that is 0.44 times the head width. The pronotum is 0.74 times the elytral width, with pronotal punctures that are unevenly distributed and 0.5–2.5 diameter apart. The elytra are similar to the pronotum and are separated by 1.0–3.0 diameters. The prosternal process (Figure 1B) is trapezoidal, with a length 2 time the basal width and with lateral margins that have distinct parallel carinae and which extend to the frontal region of the prosternum. The postcoxal lines (Figure 2D) at abdominal ventrite 1 are complete and reach 6/7ths of its length, while the male apical margins of the abdominal fifth ventrite are rounded.

Male genitalia (Figure 2E–H): the penis (Figure 2E) is stout. The penis capsule’s inner arm is distinctly long and the outer ones are short and indistinct. The penis apex (Figure. 2F) is spoon shaped with a bifurcated large membranous appendage. The tegmen (Figure 2G,H) is stout, while the penis guide along the ventral view is robust and somewhat V-shaped, curving smoothly to join the apex and with two distinct keel at the outer and inner basal areas (Figure 2G). The penis guide along the lateral view is heart-like, broadly oval-shaped and wider at the middle, converging to a blunt apex (Figure 2H). The parameres are stout and oval, shorter than the penis guide and have long setae on the apical, outer and inner margins.

Female: external appearance is similar to the male, except for abdomen ventrite 5 (Figure 2I) posteriorly which is without a median tooth and ventrite 6 which is weakly arcuate. Female spermatheca (Figure 2J) are without a nodulus and ramus.

**Figure 3:**
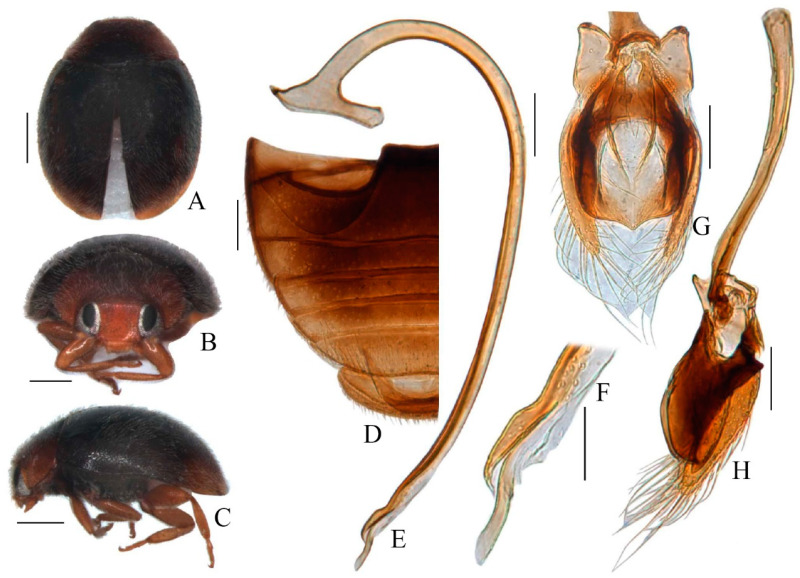
*Scymnus (Pullus) lalifolius*. (**A**) Dorsal view. (**B**) Frontal view. (**C**) Lateral view. (**D**) Abdomen. (**E**) Penis. (**F**) Apex of penis. (**G**) Ventral view of tegmen. (**H**) Lateral view of tegmen. Scale bar (mm): (**A**–**D**) 0.5; (**C**–**F**) 0.15.

Diagnosis. This species can be easily separated from *Scymnus* (*Pullus*) *latifolius* by the brownish-yellow body (Figure 2A–C), and by the broad and strongly curved apex of the penis, which forms a hook-shaped structure (Figure 2D) in the *cardi*, while in *latifolius* the apex is also hook-shaped but is not strongly curved (Figure 3D). The penis guide along the lateral view is bulbous-like in *latifolius* (Figure 3E). In *cardi* the penis guide in the ventral view is somewhat V-shaped (Figure 2F) but is U-shaped and shovel-like in *latifolius* (Figure 3F). The penis guide along the lateral view is heart-like in *cardi* (Figure 2E). The parameres are slightly shorter than the penis guide in *cardi* (Figure 2E,F), while the opposite is true for *latifolius* (Figure 3E,F).

Prey. This species predates *Myzus persicae* (Sulzer) and *Aphis gossypii* Glover (Hemiptera: Aphididae) (current study).

Host Plant. This species was collected from *Ziziphus nummularia* (Burman) (Rosales: Rhamnaceae) (current Study).

Distribution. Pakistan: Chakwal (Kallar Kahar).

### 3.2. The Mitochondrial Genome

**Figure 4:**
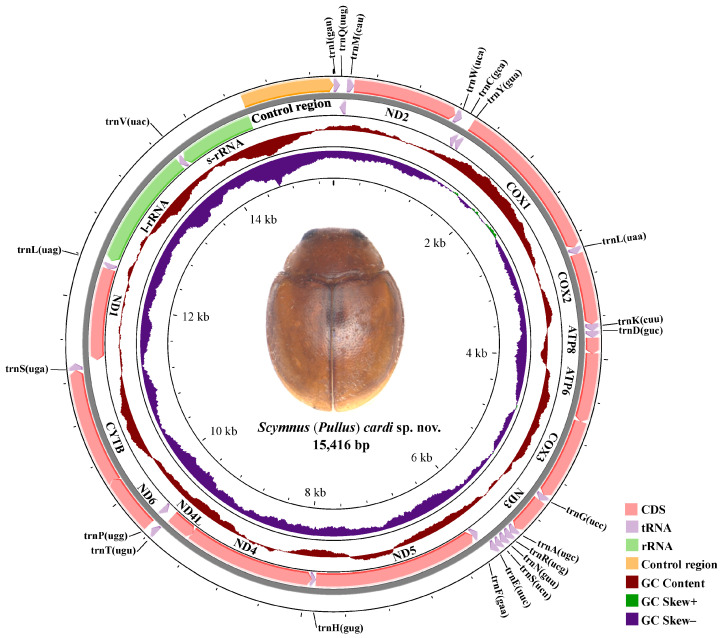
Circle maps of the mitochondrial genomes of *Scymnus* (*Pullus*) *cardi* sp. nov. Different genes are distinguished with different colors.

Genome organization. The mitogenome of *Scymnus* (*Pullus*) *cardi* (GenBank: PP639204) is 15416 bp in length, with an A + T content of 78.5%. As with other beetle mitogenomes, the nucleotide composition of the *S.* (*P.*) *cardi* has an obvious A + T bias (Table 1). The newly sequenced mitogenome contains 37 genes (13 PCGs, 2 rRNAs, and 22 tRNAs) and 1 control region (876 bp, A + T ratio 85.1%). Nine of the PCGs and 14 tRNAs are encoded on the positive strand (forward strand) and the remaining 4 PCGs, 8 tRNAs, and 2 rRNAs are located on the negative strand (reverse strand) (Table S1 and Figure 4).

The gene arrangement of the new mitogenome is similar to those of the other Coccinellidae mitochondrial genomes, studied by Kim et al. [74], Behere et al. [64], Niu et al. [60], Seo et al. [53], Hao et al. [61], Zhou et al. [62], Salazar and Nattier [59] and Zhang et al. [65], and contain the typical set of mitochondrial genes present in insects.

**Figure 5:**
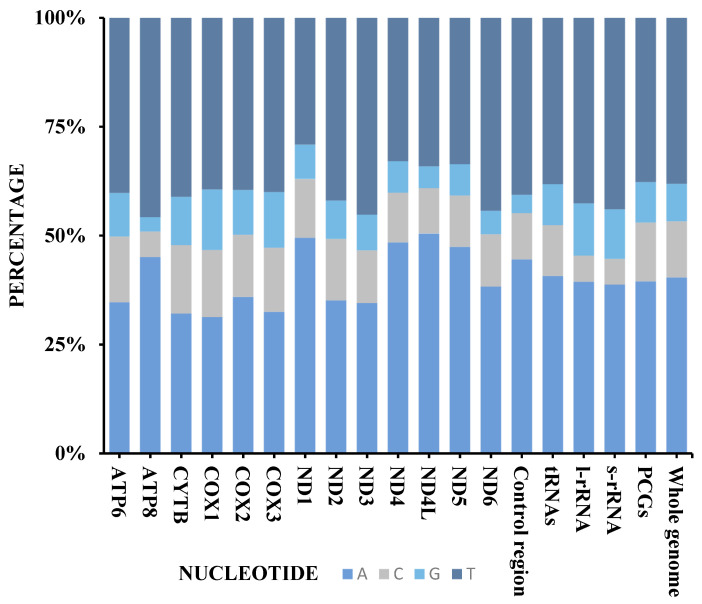
Nucleotide composition of the *Scymnus* (*Pullus*) *cardi* sp. nov. mitogenome. Protein-coding genes (PCGs), transfer RNAs (tRNAs), ribosomal RNAs (rRNAs), and the control region.

The general composition of the GC/AT ratio of the *Scymnus* (*Pullus*) *cardi* mitogenome is 21.5/78.5%, PCGs 22.8/77.2%, rRNAs 17.8/82.1%, and tRNAs 21.1/78.9%, with negative GC skew and positive AT skew (Table S2 and Figure 5). The AT skew and GC skew of 13 PCGs in *S.* (*P.*) *cardi* range from 0.26 (ND1) to −0.13 (ND3) and −0.05 (COX1) to −0.38 (ND6).

PCGs, rRNAs, tRNAs, and control region. The total length of the 13 PCGs of *Scymnus* (*Pullus*) *cardi* is 11,060 bp and begins with the typical mitogenome codon ATN (N represents C, G, and T), except for the ND2 gene with AAT, and the ND1, ND4 and ND6 genes with TAT. All stop codons of the 13 PCGs were TAA/TAG or incomplete stop codons by T, except ND1 with CTG (Table S1), which are observed in the mitogenomes of beetles [59,62,75].

The large ribosomal RNA (16S rRNA) of *Scymnus* (*Pullus*) *cardi* is 1276 bp in size, with an A + T content of 82%, and its small ribosomal RNA (12S rRNA) is 793 bp, with an A + T content of 82.6% (Tables S1 and S2 and Figure 5). The two rRNA genes are located between tRNA-Leu1 and tRNA-Val and tRNA-Val and the control region, meaning that i resembles the mitogenome of previously studied ladybird beetles [59,62,65].

The 22 tRNAs have a size range of 57 bp (trnS1) to 70 bp (trnK) and are of 1413 bp in total length. All of the tRNA present a canonical cloverleaf secondary structure with the conventional four arms, except the trnS1 (tRNA-Serine1), which lacks the D-stem or loop in the dihydrouridine (DHU) arm, as in many beetle species (Figure S1). The tRNAs that include trnW, trnE, trnH, and trnP have smaller T-loop motifs. The tRNAs that include trnQ, trnC, trnA, trnR, trnV, and trnS2 have smaller D-loop motifs (Figure S1). The length of the trnS1 genes in *Scymnus* (*Pullus*) *cardi*, *Scymnus* (*Pullus*) *loewii,* and *S*. (*P*.) *rubricaudus* range from 57 bp to 55 bp and all these species have a UCUs codon in the anticodon loop (AC loop) (Figure S4), as in many ladybird beetle species [76]. The structure of the UCUs in the anticodon loop might be considered to be indicative of those of more ancient insect species [77].

The control region (A + T-rich region) of *Scymnus* (*Pullus*) *cardi* is located between the 12S rRNA and trnI–trnG–trnM cluster (Tables S1 and S2 and Figure 5) with a high A + T content of 86% (Tables S1 and S2, Figure 5).

Codon Usage. The relatively synonymous codon usage (RSCU) values for the PCGs in the mitogenome of the *Scymnus* (*Pullus*) *cardi* were analyzed and compared here with *Scymnus* (*Pullus*) *loewii,* and *Scymnus* (*Pullus*) *rubricaudus* and showed very high similarity. A and U were more frequently used than G and C. The four most frequently used codons in three species of genus *Scymnus* are UUA (Leu2), UCU (Ser2), CGA (Agr), and GGA (Gly) (Figure S3).

### 3.3. Phylogenetic Analyses

**Figure 6:**
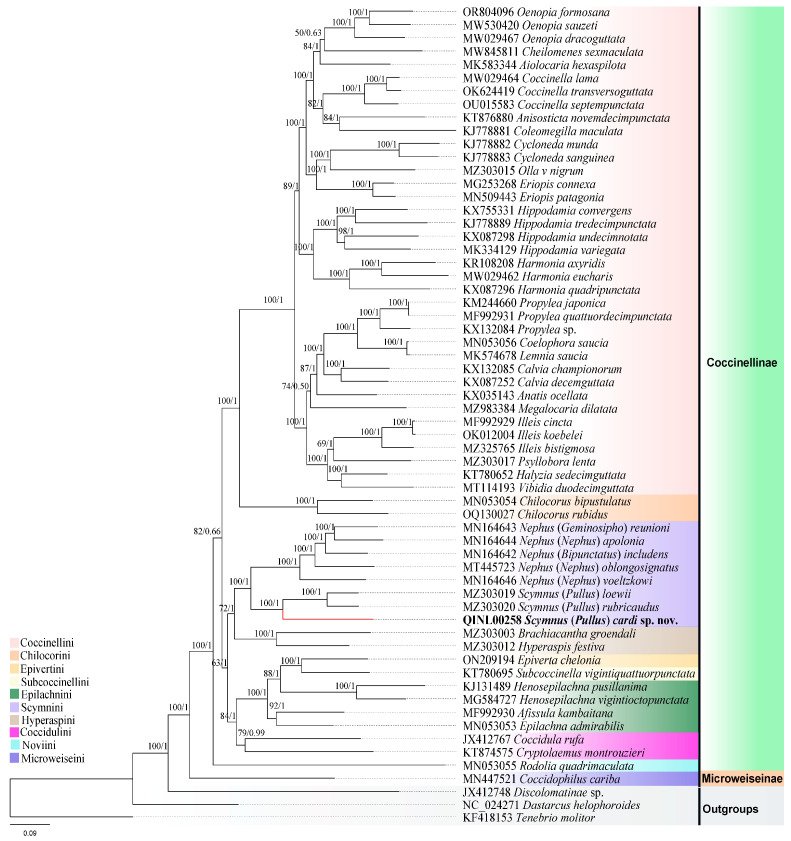
Phylogenetic relationships between *S*. (*P*.) *cardi* sp. nov. (highlighted in red and the tip is bold) and 61 other beetles. The tree was inferred by the maximum likelihood (ML) method based on 13 PCGs and 2 ribosomal RNAs (13 PCGs + 2rRNAs). Scale bar refers to a phylogenetic distance of 0.045 nucleotide substitutions per site. Node numbers show bootstrap support values (left) and Bayesian posterior probability support values (right). Different background colors indicate different tribes.

The phylogenetic analyses of 15 mitochondrial genes from 59 ladybird species and 3 outgroups confirm that the sequenced species *Scymnus* (*Pullus*) *cardi* sp. nov. is nested in the subgenus *Pullus*, with 100% support value. Furthermore, our results indicate that the genus *Scymnus* is the sister group of *Nephus*, with a high support value (BS = 100%, PP = 1) (Figure 6). These results were recovered in all concatenated datasets using maximum likelihood (ML) and Bayesian inference (BI) analyses.

Based on the 13 PCGs and 2 rRNAs datasets, the phylogenetic trees were reconstructed by 2 inference methods (ML and BI). The BI trees and ML analyses presented the same topology (Figure 6). The results exhibit a strongly resolved relationship between tribes, with high posterior probability support values (PP = 1), except for *Cheilomenes* + *Oenopia* (PP = 0.63), and *Megalocaria* + *Anatis* (*Calvia* + *Lemnia* + *Coelophora* + *Propylea*) (PP = 0.50) in tribe Coccinellini.

The 13 PCGs_AA partition scheme produced a slightly different topology in the BI tree, with *Anatis* found to be sister to *Megalocaria* (*Halyzia* + *Vibidia* + *Psyllobora* + *Illeis*) with a moderate support value (PP = 0.89), while in ML, *Megalocaria* was not recovered as sister of the *Anatis* (Figure S2). Similarly, In the 13 PCGs + 2rRNAs dataset, the clade *Coccinella* was found to be a sister to *Aiolocaria* + *Cheilomenes* with a high support value (BS = 89%, BL = 1), which is not observed in the other datasets (13 PCGs_AA). In the 13 PCGs + 2rRNAs dataset, both ML and BI analyses place the tribe Novini as the basal group in the subfamily Coccinellinae (Figure 6), whereas, in the 13 PCGs_AA dataset, both ML and BI analyses supported the tribe Coccidulini together with Epilachnini, which diverges as an early sister group in Coccinellinae (Figure S2).

## 4. Discussion

In the present study, we describe the new species *Scymnus* (*Pullus*) *cardi* of *Scymnus* and provide its mitochondrial genome, which gives comprehensive information for the identification of this species.

*Scymnus* (*Pullus*) *cardi* is allied with the clade of *Scymnus* (*Pullus*) *latifolius* and *Scymnus* (*Pullus*) *loewii* nested in subgenus *Pullus*. Based on the brief description of *S.* (*P*.) *latifolius* by Poorani and Lalitha [78], the most relevant characteristics that distinguish these species are the body appearance and male genitalia. *S.* (*P*.) *cardi* has a yellowish-brown body, while *S.* (*P*.) *latifolius* has a dark brown body with an elytral discal area of yellow to light brown. The male genitalia of *S.* (*P*.) *cardi* has a spoon-shaped structure at the penis apex, and a V-shaped structure of the penis guide in the ventral view, whereas, in *S.* (*P*.) *latifolius* the penis apex has a weak and slight curve, and the penis guide in the ventral view is U shaped. These distinct characteristics of the male genitalia of *S.* (*P*.) *cardi* differentiate this new species from the six recorded species of the subgenus *Pullus* in Pakistan [20,26,79,80]. The type locality of *S*. (*P*.) *cardi* is Kot Sarang, located in district Chakwal of Punjab province. The type specimen of *S*. (*P*.) *cardi* was collected from the *Ziziphus nummularia* plants of the family Rhamnaceae and was found feeding on *Myzus persicae* (Aphididae), while the paratypes collected from Kallar Kahar (Chakwal) were found feeding on both *Aphis gossypii* and *Myzus persicae* (Aphididae) of *Ziziphus nummularia*. Specimens of *S*. (*P*.) *latifolius* were collected from district Rawalakot (33°40.668′ N, 73°34.456′ E, 478 m) of Azad Jammu and Kashmir. Previous studies have indicated that most of the reported Pakistani species of the subgenus *Pullus* feed on members of the families Apididae and Pseudococcidae [79,80]. According to the paper of Poorani and Lalitha [78], *S*. (*P*.) *latifolius* predates on three species of the family Pseudococcidae (*Ferrisia virgata*, *M. hirsutus*, and *Paracoccus marginatus*) of mulberry and eggplant in India.

To provide more genetic information about the new species, its mitochondrial genome was obtained using next-generation sequencing. Based on 59 taxa and 3 outgroups, we explored the phylogenetic relationships of Coccinellidae using two different methods (IQ-TREE and Phylobayes) based on two datasets (13 PCGs+2r RNAs, and 13 PCGs_AA), which recovered this family into two main clades (Coccinellinae and Microweiseinae). These results strongly support the placement of the new species *S*. (*P*.) *cardi* in the genus *Scymnus* Kugelann, 1794, with a high support value (BS = 100%, PP = 1) (Figure 6).

Che et al. [13] conducted a comprehensive study on the phylogenetic relationships within the family Coccinellidae, providing strong support for the notion of the monophyly of several tribes within the subfamily Coccinellinae. Their research consistently found Coccinellini to be monophyletic, which contrasts with previous studies [11,55,57,76,81,82] that have suggested it to be the sister group of Chilocorini. Our results also recovered a similar branching pattern showing Coccinellini as a sister to Chilocorini based on the mitogenome data (Figure 6).

The tribes Chilocorini, Epilachnini, Hyperaspini, Scymnini, and Coccidulini are often considered paraphyletic or polyphyletic [57,82]. Our mitochondrial genome data supported a sister-group relationship between Coccidulini and the clade Epilachnini (Epivertini + Subcoccinellini), consistent with the mitochondrial sequence findings of Zhang et al. [65]. However, Scymnini and Hyperaspini were found to be paraphyletic in our analyses, which was also observed in previous studies [11,13,55,82].

The monophyly of Coccinellidae and several genera within it were supported here. Our study suggests that increased taxa sampling is necessary to comprehensively evaluate the phylogenetic relationships of Coccinellidae in the future.

## 5. Conclusions

The combination of morphological and mitogenomic data allows us to describe a new species of family Coccinellidae, *Scymnus* (*Pullus*) *cardi*, and identify its phylogenetic relationship. In this study, we utilized high-throughput sequencing technology to reconstruct the mitochondrial genome *S*. (*P*.) *cardi*, which is 15416 bp in length and expresses high AT bias. Our phylogenetic analysis revealed the phylogenetic position of *S*. (*P*.) *cardi* and indicated that it belongs to the genus *Scymnus* in the tribe Scymnini. The *S*. (*P*.) *cardi* mitogenome reported here adds to the number of Coccinellidae mitochondrial genomes available for future work. Additionally, our sequenced mitogenome with morphology was helpful in the identification of *S*. (*P*.) *cardi.* This study will contribute to future work on population genetics and the evolution of the genus *Scymnus*

## Data Availability

The following information was supplied regarding the availability of DNA sequences: the new mitogenomes are deposited in GenBank of NCBI and the accession numbers are PP639204.

## Supplementary Materials

The following supporting information can be downloaded at: https://www.mdpi.com/article/10.3390/insects15050371/s1, Figure S1: The predicted secondary structures of 22 tRNA genes inferred for *Scymnus* (*Pullus*) *cardi* sp. nov. The red circle dot indicates a mismatched base, and the blue line represent a matched base; Figure S2. Phylogenetic relationships between *S*. (*P*.) *cardi* sp. nov. (highlighted in red and the tip is bold) and 61 other beetles. Tree inferred by maximum likelihood (ML) method based on 13 PCGs being translated into amino acids (13 PCG_AA). Scale bar refers to a phylogenetic distance of 0.045 nucleotide substitutions per site. Node numbers show bootstrap support values (left) and Bayesian posterior probability support values (right). Different background colors indicate different tribes. (--) indicates that the node is not recovered by BI analysis; Figure S3: Relative synonymous codon usage (RSCU) of *Scymnus* (*Pullus*) *cardi* sp. nov., *Scymnus* (*Pullus*) *loewii*, and *Scymnus* (*Pullus*) *rubricaudus* mitochondrial genomes; Figure S4: The predicted secondary cloverleaf structure for the trn-Ser1 genes of *Scymnus* (*Pullus*) *cardi* sp. nov., *Scymnus* (*Pullus*) *loewii,* and *Scymnus* (*Pullus*) *rubricaudus*. Table S1: Summary of the mitogenome of *Scymnus* (*Pullus*) *cardi* sp. nov.; Table S2: Nucleotide composition of the *Scymnus* (*Pullus*) *cardi* sp. nov. mitogenome. Protein-coding genes (PCGs), ribosomal RNAs (rRNAs), and transfer RNAs (tRNAs).
