# Supplementary material for: A New Species of Scymnus (Coleoptera, Coccinellidae) from Pakistan with Mitochondrial Genome and Its Phylogenetic Implications
Source: Insects. 2024 May 19;15(5):371. doi: 10.3390/insects15050371 (PMC11122443; doi:10.3390/insects15050371)
Supplement: Supplementary file 1 [file insects-15-00371-s001.zip › Supplementary data file-Insects.pdf]

Table S1. Summary of the mitogenome of *Scymnus* (*Pullus*) *cardi* sp. nov.

Genes	Position	Length (bp)	Start Codon	Stop codon	Anticodon	Direction	Intergenic Nucleotide
trnI	1-62	62			GAT	Forward	0
trnQ	62-127	66			TTG	Reverse	-2
trnM	129-197	69			CAT	Forward	-1
ND2	198-1199	1002	AAT	TAA		Forward	-4
trnW	1203-1270	68			TCA	Forward	7
trnC	1263-1323	61			GCA	Reverse	-2
trnY	1325-1387	63			GTA	Reverse	-2
COX1	1389-2921	1533	ATA	TAG		Forward	-3
trnL2	2924-2987	64			TAA	Forward	-1
COX2	2988-666	679	ATA	TAG		Forward	-1
trnK	3667-3736	70			CTT	Forward	0
trnD	3736-3800	65			GTC	Forward	-1
ATP8	3801-3953	153	ATT	TAG		Forward	6
ATP6	3947-4607	661	ATG	TAA[1]		Forward	-1
COX3	4608-5388	781	ATG	TAA[1]		Forward	-1
trnG	5389-5452	64			TCC	Forward	-1
ND3	5453-5806	354	ATT	TAG		Forward	1
trnA	5805-5867	63			TGC	Forward	0
trnR	5867-5931	65			TCG	Forward	2
trnN	5929-5993	65			GTT	Forward	-1
trnS1	5994-6050	57			TCT	Forward	-1
trnE	6051-6113	63			TTC	Forward	1
trnF	6112-6175	64			GAA	Reverse	-1
ND5	6176-7890	1715	ATT	TAA		Reverse	-1
trnH	7891-7952	62			GTG	Reverse	-1
ND4	7953-9270	1318	TAT	TAA		Reverse	3
ND4L	9267-9551	285	ATA	TTA		Reverse	-1
trnT	9552-9616	65			TGT	Forward	-1
trnP	9617-9679	63			TGG	Reverse	-3
ND6	9682-10184	503	TAT	TAA		Forward	0
CYTB	10184-11311	1128	ATA	TAA		Forward	1
trnS2	11310-11375	66			TGA	Forward	-18
ND1	11393-12340	948	TAT	CTA		Reverse	-4
trnL1	12344-12405	62			TAG	Reverse	-1
l-rRNA	12406-13682	1276				Reverse	-1
trnV	13683-13748	66			TAC	Reverse	0
s-rRNA	13748-14540	793				Reverse	-1
Control region	14541-15416	876				-	

The exponent numerals in the gene column are used to differentiate each of the two Leucine- and Serine-specifying transfer RNAs (tRNAs) (L1 and L2, S1 and S2); TAA[1] stop codon is completed by the addition of 3′ A residues to mRNA; (T–) stop codon not determined.

Table S2. Nucleotide composition of the *Scymnus* (*Pullus*) *cardi* sp. nov. mitogenome. Protein-coding genes (PCGs), ribosomal RNAs (rRNAs), and transfer RNAs (tRNAs).

Feature	Proportion (%)						Skew		Nucleotide (bp)
	A	C	G	T	A +T	G + C	AT	GC	
**Whole genome**	40.4	12.9	8.6	38.1	78.5	21.5	0.03	-0.20	**15,416**
**PCGs**	39.5	13.5	9.3	37.7	77.2	22.8	0.02	-0.18	**11,060**
**ATP6**	34.7	15.1	10	40.2	74.9	25.1	-0.07	-0.20	153
**ATP8**	45.1	5.9	3.3	45.8	90.9	9.2	-0.01	-0.28	661
**CYTB**	32.1	15.7	11.1	41.1	73.2	26.8	-0.12	-0.17	1,128
**COX1**	31.3	15.4	13.9	39.4	70.7	29.3	-0.11	-0.05	1533
**COX2**	35.9	14.3	10.3	39.5	75.4	24.6	-0.05	-0.16	679
**COX3**	32.5	14.7	12.8	40	72.5	27.5	-0.10	-0.07	781
**ND1**	49.5	13.5	7.9	29.1	78.6	21.4	0.26	-0.26	948
**ND2**	35.1	14.1	8.8	41.9	77	22.9	-0.09	-0.23	1,002
**ND3**	34.5	12.1	8.2	45.2	79.7	20.3	-0.13	-0.19	354
**ND4**	48.4	11.4	7.2	32.9	81.3	18.6	0.19	-0.23	1318
**ND4L**	50.5	10.4	5	34.1	84.6	15.4	0.19	-0.35	285
**ND5**	47.4	11.8	7.2	33.6	81	19	0.17	-0.24	1,715
**ND6**	38.3	12.0	5.4	44.3	82.6	17.4	-0.07	-0.38	503
**rRNAs**	43.1	11.8	6	39.0	82.1	17.8	0.05	-0.33	**2,069**
**l-rRNA**	39.4	6	12	42.6	82	18	-0.04	0.33	1276
**s-rRNA**	38.7	5.9	11.4	43.9	82.6	17.3	-0.06	0.32	793
**tRNAs**	40.7	11.7	9.4	38.2	78.9	21.1	0.03	-0.11	**1,413**
**Control region**	44.5	10.6	4.2	40.6	85.1	14.8	0.05	-0.43	**876**

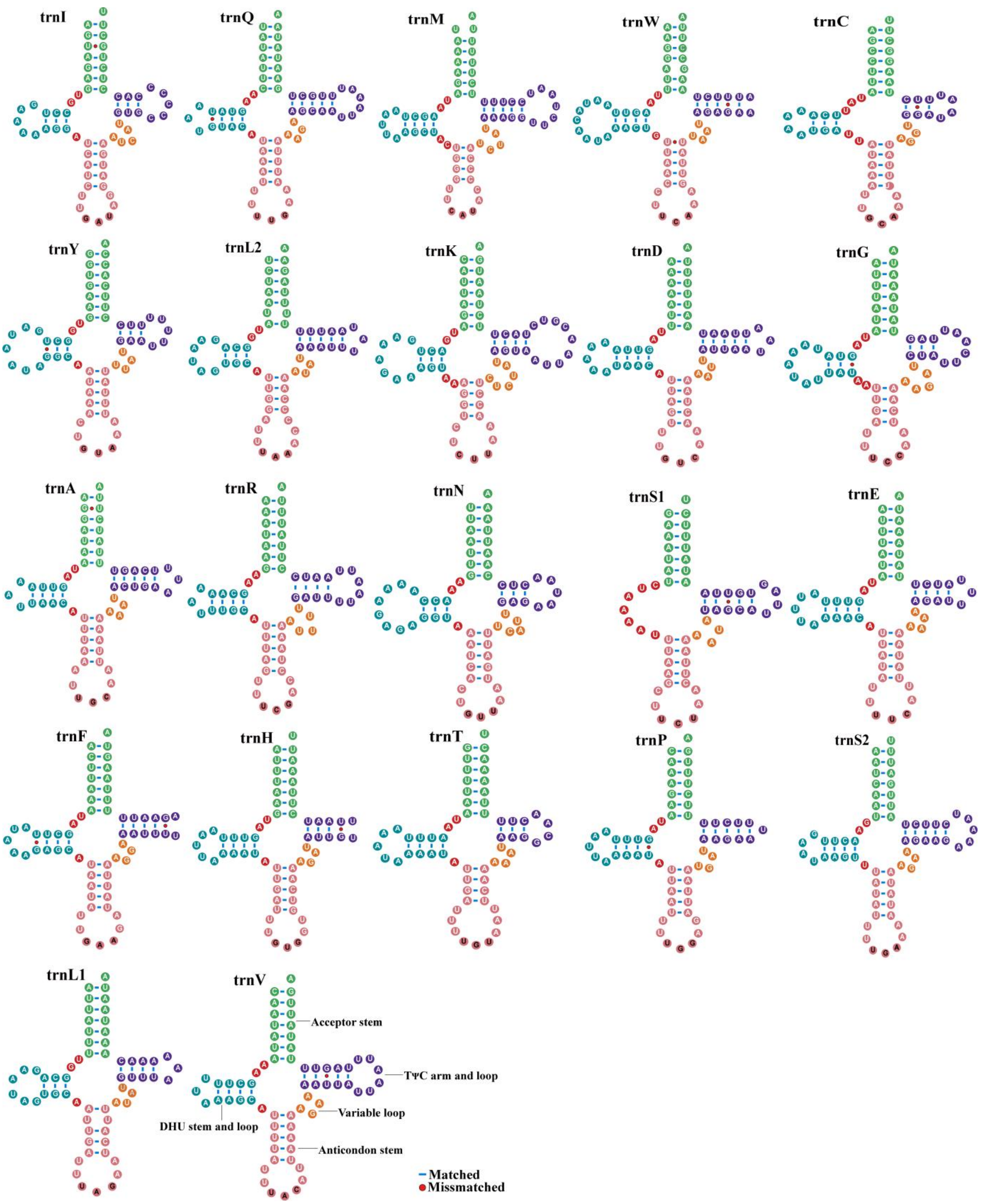

Figure S1. The predicted secondary structures of 22 tRNA genes inferred for *Scymnus* (*Pullus*) *cardi* sp. nov. The red circle dot indicates a mismatched base, and the blue line represents a matched base.

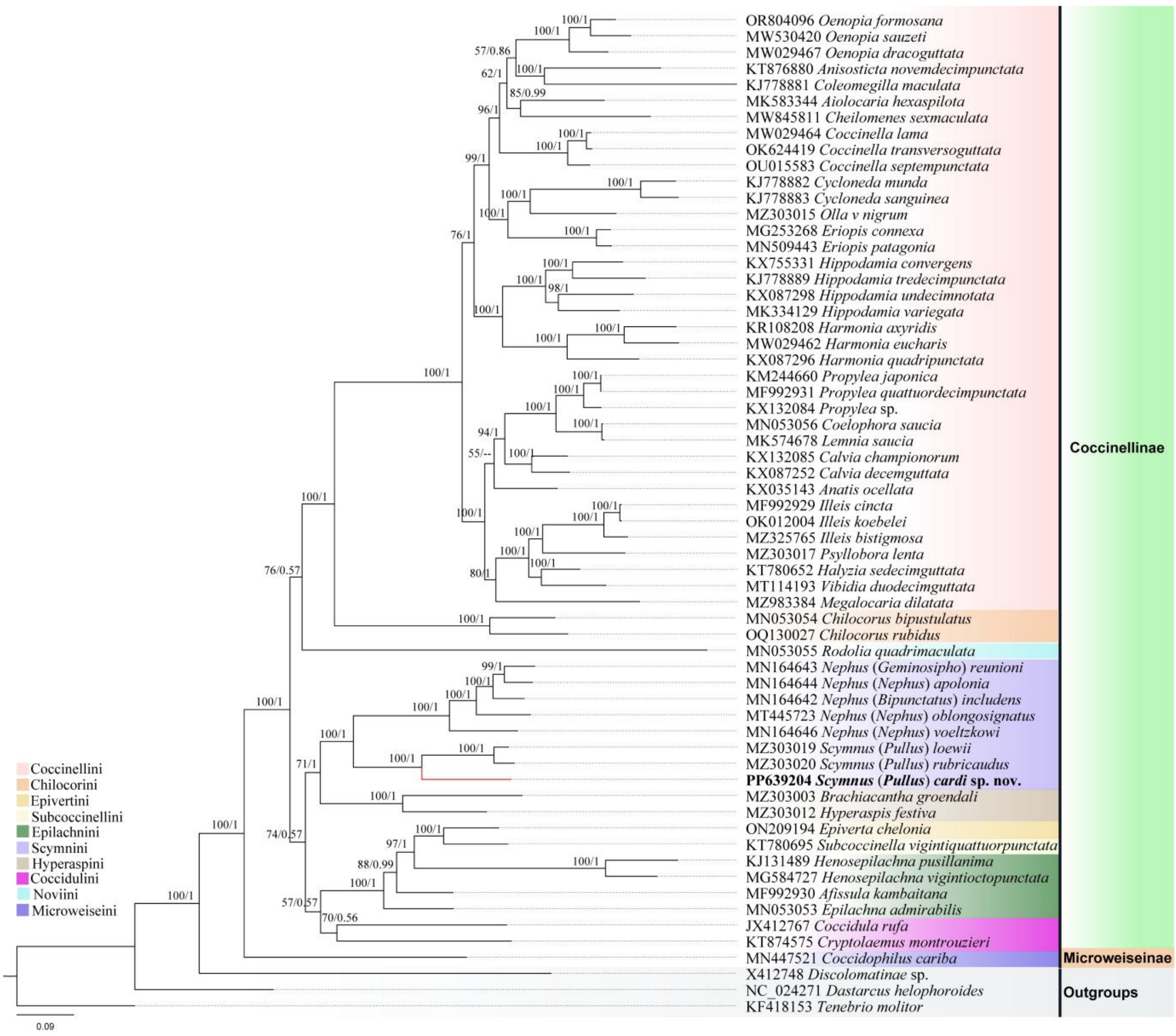

Figure S2. Phylogenetic relationships between S. (*P.*) *cardi* sp. nov. (highlighted in red and the tip is bold) and 61 other beetles. Tree inferred by maximum likelihood (ML) method based on 13 PCGs being translated into amino acids (13 PCG_AA). Scale bar refers to a phylogenetic distance of 0.045 nucleotide substitutions per site. Node numbers show bootstrap support values (left) and Bayesian posterior probability support values (right). Different background colors indicate different tribes. (--) indicates that the node is not recovered by BI analysis.

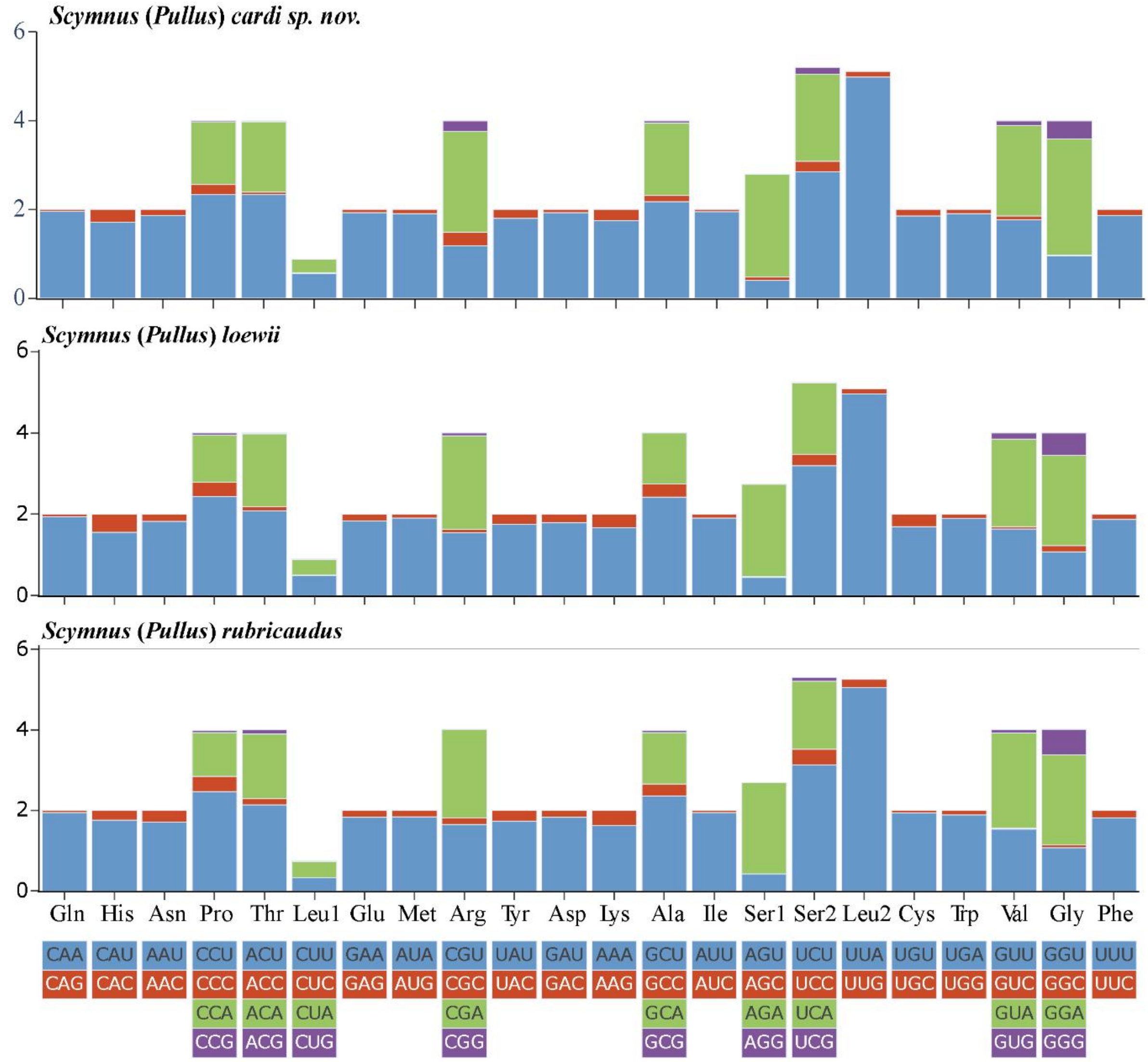

Figure S3. Relative synonymous codon usage (RSCU) of *Scymnus* (*Pullus*) *cardi* sp. nov., *Scymnus* (*Pullus*) *loewii*, and *Scymnus* (*Pullus*) *rubricaudus* mitochondrial genomes.

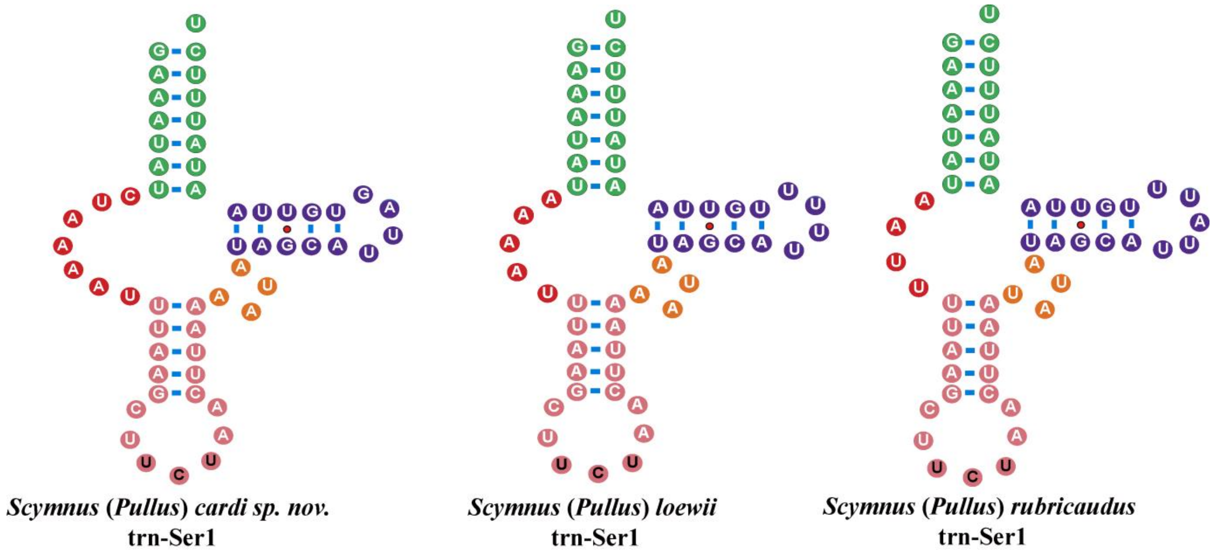

Figure S4. The predicted secondary cloverleaf structure for the trn-Ser1 genes of *Scymnus* (*Pullus*) *cardi* sp. nov., *Scymnus* (*Pullus*) *loewii,* and *Scymnus* (*Pullus*) *rubricaudus*.
